# Dexamethasone prophylaxis protects from acute high-altitude illness by modifying the peripheral blood mononuclear cell inflammatory transcriptome

**DOI:** 10.1042/BSR20231561

**Published:** 2023-11-30

**Authors:** Rahul Kumar, Neha Chanana, Kavita Sharma, Tsering Palmo, Michael H. Lee, Aastha Mishra, Kevin Nolan, Dara C. Fonseca Balladares, Claudia Mickael, Mohit D. Gupta, Tashi Thinlas, Qadar Pasha, Brian B. Graham

**Affiliations:** 1 Department of Medicine, University of California San Francisco, San Francisco, CA, U.S.A.; 2 Lung Biology Center, Zuckerberg San Francisco General Hospital, San Francisco, CA, U.S.A.; 3 Genomics and Molecular Medicine, CSIR-Institute of Genomics and Integrative Biology, Delhi, India; 4 Cardiorespiratory Disease Unit, CSIR-Institute of Genomics and Integrative Biology, Delhi, India; 5 Division of Pulmonary Sciences and Critical Care Medicine, Department of Medicine, University of Colorado Anschutz Medical Campus, CO, U.S.A.; 6 Department of Cardiology, GB Pant Institute of Post Graduate Medical Education and Research, New Delhi, India; 7 Department of Medicine, Sonam Norboo Memorial Hospital, Leh, Ladakh, India; 8 Institute of Hypoxia Research, New Delhi, India

**Keywords:** High-altitude, hypoxia, inflammation, Peripheral blood mononuclear cells, pulmonary hypertension

## Abstract

Acute high-altitude (HA) exposure can induce several pathologies. Dexamethasone (DEX) can be taken prophylactically to prevent HA disease, but the mechanism by which it acts in this setting is unclear. We studied the transcriptome of peripheral blood mononuclear cells (PBMCs) from 16 subjects at low altitude (LA, 225 m) and then 3 days after acute travel to HA (3500 m) during the India-Leh-Dexamethasone-Expedition-2020 (INDEX2020). Half of the participants received oral DEX prophylaxis 4 mg twice daily in an unblinded manner, starting 1 day prior to travel to HA, and 12 h prior to the first PBMC collection. PBMC transcriptome data were obtained from 16 subjects, half of whom received DEX. The principal component analysis demonstrated a clear separation of the groups by altitude and treatment. HA exposure resulted in a large number of gene expression changes, particularly in pathways of inflammation or the regulation of cell division, translation, or transcription. DEX prophylaxis resulted in changes in fewer genes, particularly in immune pathways. The gene sets modulated by HA and DEX were distinct. Deconvolution analysis to assess PBMC subpopulations suggested changes in B-cell, T-cell, dendritic cell, and myeloid cell numbers with HA and DEX exposures. Acute HA travel and DEX prophylaxis induce significant changes in the PBMC transcriptome. The observed benefit of DEX prophylaxis against HA disease may be mediated by suppression of inflammatory pathways and changing leukocyte population distributions.

## Introduction

Acute high-altitude (HA) environmental exposure can cause several pathologies, including acute mountain sickness (AMS), pulmonary hypertension (PH), HA pulmonary edema (HAPE), and HA cerebral edema (HACE). Chronic HA exposure can progress to chronic mountain sickness (CMS) and PH becoming more severe and irreversible with descent to low altitude (LA). Inflammation has emerged as a pivotal trigger in PH pathogenesis, particularly within low oxygen environments [1–3]. The interplay between immune cells, inflammatory cytokines, and endothelial dysfunction fuels pathogenic processes including vascular remodeling, vasoconstriction, and abnormal cell proliferation [4]. High-altitude exposure significantly influences blood gene activity, especially in the case of immune-related genes [5]. An accumulating body of evidence suggests circulating leukocytes contribute to hypoxic PH, including the recruitment of monocytes, dendritic cells (DCs), and T cells to the lung tissue, followed by their local activation [2,6]. However, the precise role of the intravascular leukocytes is not well defined in the context of HAPH. Prior studies have reported the up-regulation of several inflammation-related genes in the peripheral blood following acute HA exposure [5,7], but cell-specific contributions to these inflammatory signatures were not defined. While many HA studies utilize preclinical models of normobaric hypoxia [2,8–11], understanding their human relevance requires validation with clinical data and biospecimens. Identifying mechanisms underlying HA diseases in humans will enable the development and optimization of therapeutic strategies, such as blocking underlying inflammatory drivers [12].

The potential concept of targeting inflammatory immune cells in HA diseases is supported by the therapeutic use of dexamethasone (DEX), a potent glucocorticoid [13]. DEX promotes acclimatization to HA, prevents acute HA illnesses [14], and treats HACE and HAPE, the latter representing a severe complication of HA-induced PH [15]. However, the underlying molecular mechanisms by which DEX functions in ameliorating HA pathology remain inadequately understood. We hypothesized that DEX prevents HA pathology by blocking inflammation-related molecular pathways. We herein tested this hypothesis by performing bulk RNA sequencing (RNASeq) on banked human peripheral blood mononuclear cells (PBMCs) collected from subjects at LA who then traveled to HA, half of whom received DEX prophylaxis, to uncover gene expression changes that reflect mechanisms by which HA exposure causes pathology, and how DEX may afford protection against HA-induced diseases.

## Methods

### Authorizations

The India-Leh-Dexamethasone-Expedition-2020 (INDEX2020) study protocol was approved by the human ethical committees of the Council of Scientific and Industrial Research-Institute of Genomics and Integrative Biology (CSIR-IGIB), Delhi, India, and by the Sonam Norboo Memorial (SNM) Hospital, Leh, Ladakh, India. All procedures were performed in compliance with relevant laws and institutional guidelines. Informed consent was obtained from each subject.

### Clinical protocol

The clinical data from the present study were previously published [14]. Briefly, twenty-seven age and sex-matched healthy subjects living at lowland and of Indo-Aryan ethnicity were recruited in collaboration with the CSIR-IGIB, Delhi, India, and the SNM Hospital, Leh, Ladakh, India. Subjects with chronic diseases, SARS-CoV-2 infection, pulmonary infection, pregnant status, or those unable to give informed consent were excluded from the study. The subjects were divided into two groups of 14 and 13 subjects each. We tested the effect of one dose of DEX to maximize the statistical power of this study. 14 subjects in the first group received no treatment (referred to as control). Thirteen subjects in the second group were prophylactically given DEX (referred to as DEX-p) 4 mg orally twice a day (Wockhardt Ltd, India) [14]. Both groups were clinically assessed at LA (Delhi, India, 225 m from sea level) at Govind Ballabh Pant Institute of Postgraduate Medical Education and Research and Hospital, New Delhi, and then were flown to HA (Leh, 3500 m from sea level; equivalent to 13.5% F_i_O_2_). Subjects stayed at the HA for 3 days. The subjects in the DEX-p group received DEX starting 1 day prior to the travel, and continued taking the medication for the entire time at HA.

### PBMC isolation and processing

PBMCs were obtained using the Ficoll gradient method from a cohort of 16 subjects enrolled in the INDEX2020 study. The collection process involved obtaining samples first at low altitude (LA) and then, after a span of 3 days, at high altitude (HA). Out of the 16 subjects, paired PBMC samples were collected from 6 subjects in the control group and 6 subjects in the DEX-p group, both at LA and HA, resulting in a total of 24 samples. Additionally, from the remaining 4 subjects, one unpaired PBMC sample from each time point in both groups was added, resulting in a total of 28 samples. Briefly, 8ml blood samples were drawn in EDTA-containing tubes. An equal amount of PBS was added to the blood and laid over to Ficoll-containing tubes (Cat.#17-144-02, GE Healthcare). Subsequently, the tubes were centrifuged at 300 g in swinging bucket rotors at room temperature (23°C) for 30 min, with no brakes. The buffy coat layer was collected, transferred into a 15 ml tube and washed using an equal volume of PBS. The PBMCs were pelleted by centrifuging at 300 ***g*** for 10 min at 4°C. Next, pelleted PBMCs were resuspended in freezing media (10%DMSO + 90%FCS), transferred into pre-labeled 1.8 ml cryovial tubes, and immediately placed in a −80°C freezer in Leh SNM hospital (Ladakh, India) for banking. These samples were transported in dry ice by air to CSIR-IGIB (Delhi, India), where they were processed for RNAseq.

### RNAseq processing and analysis

The cryovial tubes were retrieved from --80°C and rapidly thawed in a 37°C water bath for 2–3 min. Thawed cells were gently transferred to a 50 ml conical tube containing 8 ml of warm complete RPMI medium (RPMI-1640 medium with 10% sterile heat-inactivated FBS). The tube was then centrifuged at 300*** g*** for 5 min, and the resulting cell pellet was washed with 0.5 ml of 1× PBS + 0.04% BSA. After another centrifugation at 300 ***g*** for 5 min, the supernatant was carefully removed without disturbing the cell pellet. Subsequently, the pellets were processed for total RNA extraction using RNeasy Micro Kit (Cat.# 74004) as per the manufacturer’s protocol. RNA concentration was quantified using a Nanodrop ND-100 Spectrophotometer, and quality was assessed using a 2100 Bioanalyzer equipped with the Agilent RNA 6000 Nano Kit. RNAs meeting the criteria of a 260:280 ratio >1.5 and an RNA integrity number (RIN) ≥8 were selected for subsequent deep sequencing.

### Library preparation and sequencing

cDNA libraries were prepared from the RNA and libraries were pooled equimolarly and sequenced on a Novaseq platform, generating 60 million paired-end reads of 150 base pairs per sample. Raw data underwent quality control, followed by read alignment to a reference genome. Gene expression quantification and subsequent differential expression analysis were performed to gain insights into PBMCs’ gene expression profiles. The fastq files were uploaded into Galaxy Online, and the samples were mapped to the hg38 RefSeq mRNA transcriptome (UCSC) using the Sailfish algorithm [16], with an output of transcripts per million reads (TPM). Differential expression between groups was quantified using DESeq2 [17].

### Pathway analysis

Pathway analysis was performed using Reactome (reactome.org).

### Deconvolution analysis

The R package ‘granulator’ was used to deconvolute the bulk RNAseq data into individual cellular components using public reference profiles [18], and a curated reference PBMC dataset [19].

### Statistics

DESeq2 statistical result *P*-values were adjusted for multiple testing with the Benjamini–Hochberg procedure, which controls false discovery rate (FDR). Analysis of variance (ANOVA) with post-hoc Tukey testing and paired *t*-tests were performed as described in the figure legends. T-test *P*-values were corrected for multiple comparisons by Bonferroni correction. *P*<0.05 was considered statistically significant.

## Results

### Sample collection and initial analysis

**Figure 1:**
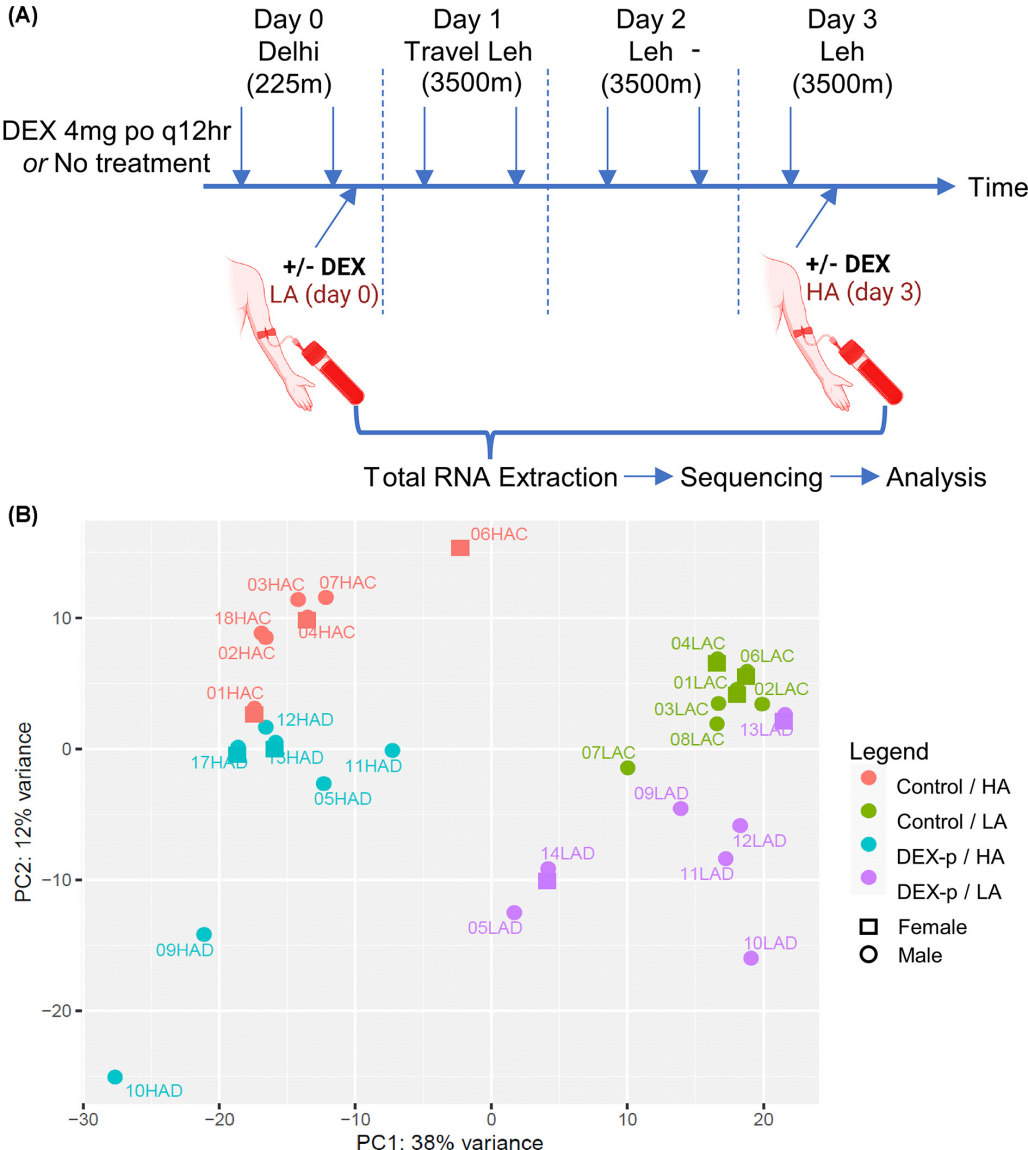
Schematic of experimental design, and principal component analysis of the PBMC samples demonstrates clustering by group (**A**) Outline of the experimental design: subjects received no treatment (control) or DEX prophylaxis (DEX-p) 4mg po q12 hours starting on Day 0, and then were flown to Ladakh on Day 1 where they resided through Day 3. Blood samples were drawn in the evening of Day 1, and mid-day on Day 3. The samples were processed for PBMCs, from which RNA was extracted and sequenced and analyzed. (**B**) Principal component analysis of the 28 PBMC samples, identified by treatment group (Control or DEX-p); altitude (low or high-altitude: LA or HA); and sex (female or male).

Banked PBMC samples, collected and banked during the collaborative INDEX2020 study, were analyzed. Results of the parent study have been published [14]. As Per the clinical protocol (Figure 1A), the subjects received DEX as prophylaxis (hereafter referred to as DEX-p) or no treatment (hereafter referred to as control), and traveled from LA to HA. We collected and processed PBMC samples from seven subjects at LA and seven subjects at HA in the control group (of which six were paired samples from the same subject), and seven subjects at LA and seven subjects at HA in the DEX-p group (also of which six were paired samples from the same subject). The demographics of these 16 participants are summarized in Table 1.

**Table 1:** Demographic data of study participants

Participants	Control (*N*=8)	DEX-p (*N*=8)
Male/Female	5/3	5/3
Age (mean ± SD)	26.4 ± 1.8	25.8 ± 1.7

*DEX-p, dexamethasone prophylaxis; N*, number of subjects; SD, standard deviation.

After processing, the transcripts were mapped to 27,070 genetic identifiers, which included coding and non-coding mRNAs. Principal component analysis (PCA) was performed on the 28 samples (Figure 1B), demonstrating strong separation of the four groups (control versus DEX-p, and LA versus HA). Of note, there was greater variance along the PC1 axis (38%) which separated the HA from LA samples, than along the PC2 axis (12%), which separated the control from DEX-p samples, suggesting a greater effect of HA exposure than DEX exposure on the PBMC transcriptome.

### Comparison of HA to LA

**Figure 2:**
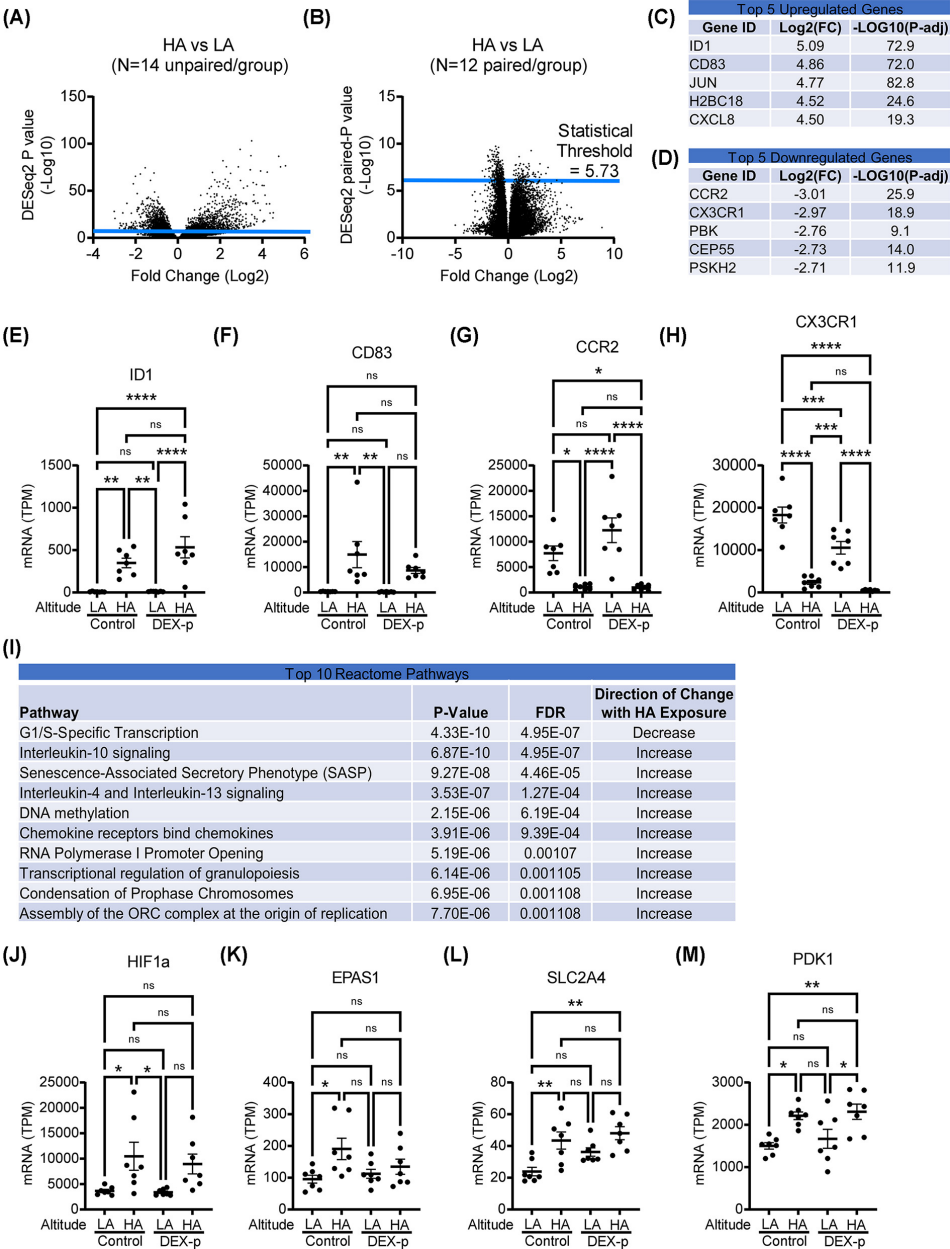
Comparison of low versus high-altitude across all samples Volcano plots, comparing fold change on the *x*-axis (log base 2) versus *P*-value (−log base 10), using (**A**) DESeq2 (*N* = 14/group) and (**B**) paired *t*-test of matched samples (*N* = 12/group); the statistical threshold corrected for multiple comparisons is shown. (**C**) Top 5 up-regulated and (**D**) top 5 down-regulated genes. Expression levels of the top 2 up-regulated genes (**E**) ID1 and (**F**) CD83, and the top 2 down-regulated genes (**G**) CCR2 and (**H**) CX3CR1 across the different groups. (**I**) Top 10 pathways by Reactome. Expression of representative prototypical hypoxia-driven genes: (**J**) HIF1a, (**K**) EPAS1/HIF2a, (**L**) SLC2A4/GLUT4, and (**M**) PDK. Gene expression units: TPM/transcripts per million. Mean ± SEM shown. N = 7/group. Post-hoc Tukey test shown *P<0.05, **P<0.005, ***P<0.001, ****P<0.0001, FDR: false discovery rate; ns: non-significant.

We identified differentially expressed sequences with altitude in the PBMCs using two approaches (of note, we use the term ‘sequence’ because we mapped subject transcriptomes against the Reference Sequence (RefSeq) database, which contains both genes and non-coding transcript sequences such as long non-coding RNAs-lncRNAs). In the first approach, the 14 HA samples were compared with the 14 LA samples, independent of control or DEX-p group, using the algorithm DESeq2 [17], which does not account for paired samples. Using a *P*-value of <0.05 adjusted for multiple comparisons, there were 2607 sequences significantly modified and either up-regulated or down-regulated at least 2-fold with HA exposure: 1975 up-regulated, and 632 down-regulated (Figure 2A). In the second approach, the 24 samples from the 12 subjects in whom both HA and LA samples were successfully drawn and processed, across both control (*n*=6) and DEX-p groups (*n*=6) were analyzed using paired *t*-test. Again, using a *P*-value of <0.05 adjusted for multiple comparisons, there were 215 sequences significantly modified and either up-regulated or down-regulated at least 2-fold: 94 up-regulated and 121 down-regulated (Figure 2B). Of these 215, only 1 of the up-regulated and 4 of the down-regulated sequences were not identified by the DESeq2 analysis. We thus decided to use the DESeq2 results with all 14 samples per group for our primary analysis.

The top 5 up-regulated and 5 down-regulated sequences (all genes) identified by DESeq2 are listed in Figure 2C,D, respectively. The top 2 up-regulated genes were *ID1* (a target of BMPR2 signaling) and *CD83* (a marker of DCs), and the distribution of expression of these genes across groups is shown in Figure 2E,F. The top 2 down-regulated genes were C-C chemokine receptor type 2 (*CCR2*) and C-X3-C motif chemokine receptor 1 *(CX3CR1*), surface receptors necessary for the recruitment of classical and non-classical monocytes, respectively, and their distribution of expression across groups is shown in Figure 2G,H.

Pathways based on gene expression differences by altitude were identified using Reactome, and the top 10 pathways are listed in Figure 2I. The pathways essentially fell into 2 categories: (1) inflammation, and (2) the regulation of cell division, translation or transcription. We also looked at the expression of several prototypical hypoxia-driven genes-hypoxia inducible factor 1 subunit alpha (*HIF1A*) (Figure 2J), endothelial PAS domain protein 1 (*EPAS1*) (Figure 2K), and the prototypical HIF1A targets glucose transporter type 4 (*GLUT4*, Figure 2L) and pyruvate dehydrogenase kinase 1 (*PDK1*, Figure 2M) to validate the anticipated biologic effect of travel to HA, and found these gene expressions were higher in the HA groups, largely independent of treatment group.

### Comparison of DEX to control

**Figure 3:**
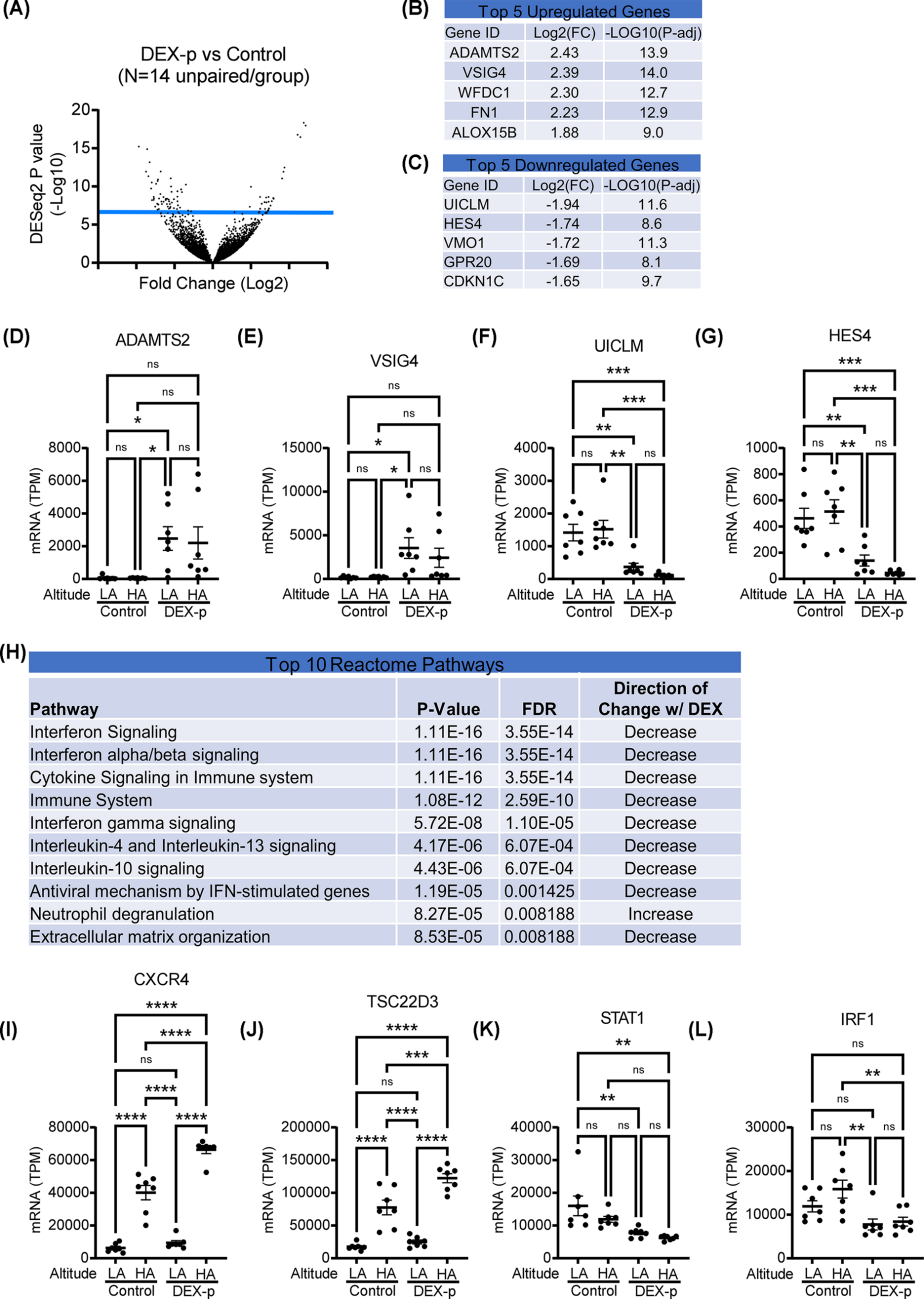
Comparison of DEX prophylaxis versus control across all samples (**A**) Volcano plot, comparing fold change on the *x*-axis (log base 2) versus *P*-value (−log base 10) by DESeq2 (*N* = 14/group). (**B**) Top 5 up-regulated and (**C**) top 5 down-regulated genes. Expression levels of the top 2 up-regulated sequences (**D**) ADAMTS2 and (**E**) VSIG4, and the top 2 down-regulated sequences (**F**) UICLM and (**G**) HES4 across the different groups. (**H**) Top 10 pathways. Expression of representative prototypical DEX-modulated genes: (**I**) CXCR4, (**J**) TSC22D3, (**K**) STAT1, and (**L**) IRF1. Expression units: TPM/transcripts per million. Mean ± SEM shown. *N* = 7/group. Post-hoc Tukey test shown *P<0.05, **P<0.005, ***P<0.001, ****P<0.0001; FDR: false discovery rate; ns: non-significant.

The 14 DEX-p samples were compared with the 14 control samples, independent of elevation at the time of blood draw. By DESeq2 analysis, with a corrected *P*-value of <0.05, there were 167 sequences up-regulated or down-regulated at least 2-fold with DEX prophylaxis: 91 up-regulated and 76 down-regulated (Figure 3A). The top 5 up-regulated and down-regulated sequences are listed in Figure 3B,C, respectively. The two most up-regulated with DEX prophylaxis were *ADAMTS2* (**D**) and *VSIG4* (**E**), and the distribution of expression of these genes across groups is shown in Figure 3D,E. *ADAMTS2* encodes the A disintegrin and metalloproteinase with thrombospondin motifs 2 protein, which processes procollagens; VSIG4 encodes the V-set and immunoglobulin domain containing four proteins, which are expressed by macrophages. The two most down-regulated were *UICLM* and *HES4* (Figure 3F,G, respectively), which encodes a lncRNA, up-regulated in colorectal cancer metastasized to the [20,21], and bHLH transcription factor 4 which is also associated with cancer [22].

The top 10 altered pathways are listed in Figure 3H: all were decreased except for 1 related to neutrophil degranulation; neutrophil degranulation has been reported to be stimulated by glucocorticoids *in vitro* [23]. Almost all of the suppressed pathways were related to immune signaling. We also looked at several prototypical DEX responsive genes, and found C-X-C chemokine receptor type 4 (*CXCR4*) (Figure 3I) and glucocorticoid-induced leucine zipper protein (*TSC22D3*; Figure 3J) were up-regulated as expected (although noted to be potentiated by concurrent HA exposure), and signal transducer and activator of transcription 1 (*STAT1*; Figure 3K) and interferon regulatory factor 1 (IRF1; Figure 3L) were down-regulated as expected [24,25].

### Interaction between DEX prophylaxis and HA exposure

**Figure 4:**
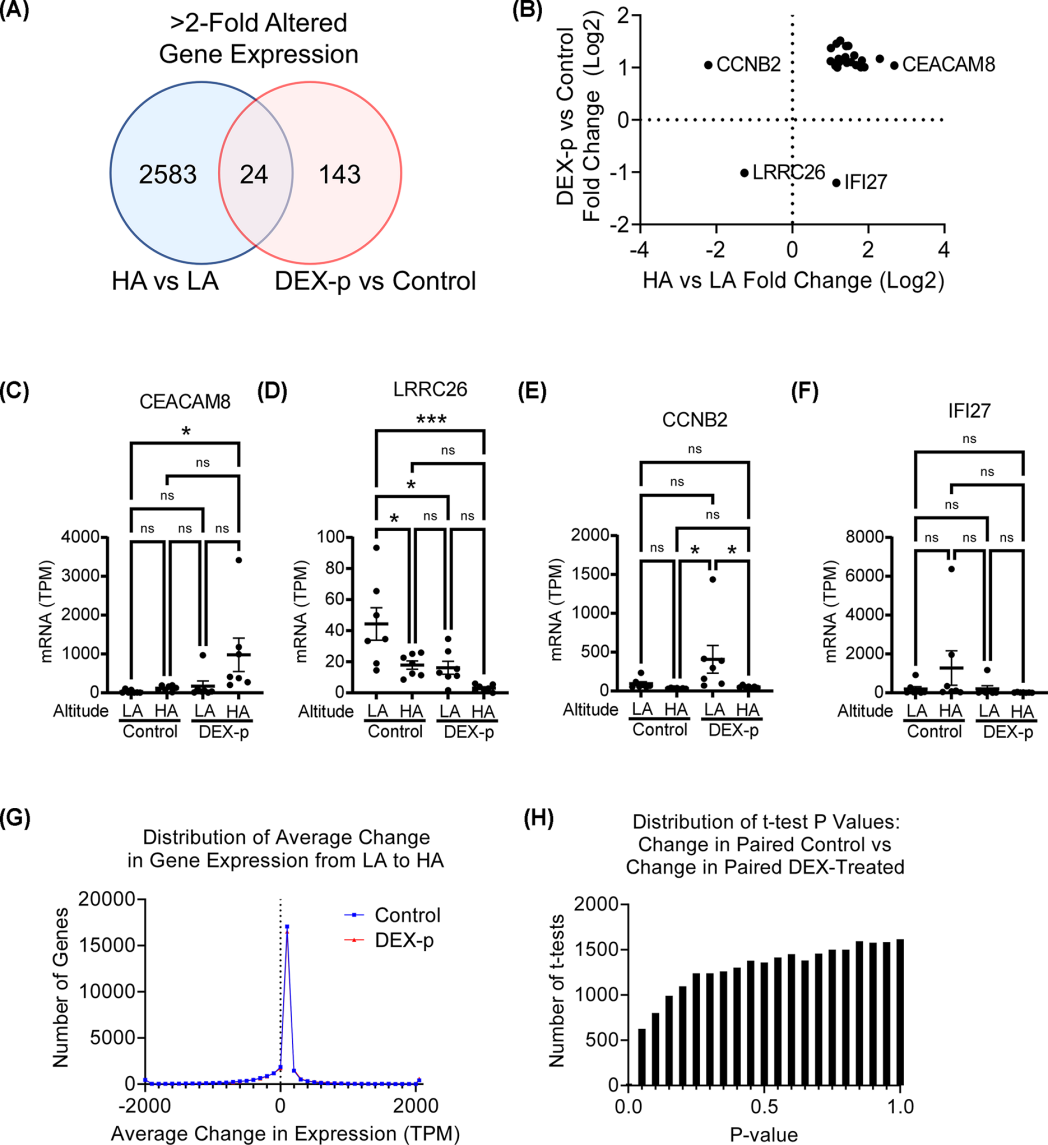
Little evidence of direct transcript-level interaction between DEX prophylaxis and HA exposure (**A**) Venn diagram showing overlap between transcripts with >2 fold change with change in elevation, and >2 fold change with DEX prophylaxis. (**B**) Of the 24 sequences identified, the direction of change with HA exposure and DEX prophylaxis. (**C–F**) Representative expressions of genes in the 4 quadrants of panel (B), CEACAM8, LRRC26, CCNB2, and IFI27 (mean ± SEM shown; *N* = 7/group; post-hoc Tukey test shown *P<0.05, **P<0.005, ***P<0.001, ****P<0.0001). (**G**) Histogram of the average change in sequence expression in paired samples from six control and six DEX-p subjects upon travel from LA to HA. (**H**) Histogram of *t*-test *P*-values for each transcript, between the changes in expression on traveling from LA to HA between the six paired control, and the six paired DEX-treated subjects.

To test the concept that DEX could counteract the HA-induced transcription signature in PBMCs, we looked for interactions between DEX prophylaxis and HA exposure using three complementary approaches. The first approach was comparing the sequence set lists modulated by each intervention. Of the 2607 sequences modulated at least 2-fold by HA exposure, and the 167 sequences modulated at least 2-fold by DEX prophylaxis, there were only 24 shared by both lists (Figure 4A). Of these 24, 21 had a positive synergistic effect between DEX and HA exposures (Figure 4B). A representative example of these is CEACAM8, encoding the cell adhesion protein CD66b (Figure 4C), which was minimally increased by either HA alone or DEX alone, whereas the combined impact of both increased *CEACAM8* expression significantly. In contrast, leucine rich repeat containing 26 (*LRRC26*, a tumor suppressor [26]) was modestly decreased by either stimulus alone, whereas the combined stimuli decreased *LRRC26* expression significantly (Figure 4D). The two genes *CCNB2* (cyclin B2, regulating cell proliferation) and IFI27 (a promoter of epithelial-mesenchymal transition [27]), which had a discordant expression, were actually little impacted by either stimulus (Figure 4E,F, respectively).

The second approach calculated the change in expression of each of the 27,070 identified sequences in the paired samples from the six control subjects who went from LA to HA, and the 6 DEX-p subjects who went from LA to HA. We plotted the average of these gene expression changes across the six control subjects and the six DEX-p subjects, and observed that the two curves were nearly superimposable, indicating that in aggregate the paired samples were not impacted by DEX prophylaxis (Figure 4G). The third approach was taking the paired change in expression with HA challenge in each subject and performing a *t*-test between the six control samples and the six DEX-p samples. We then plotted the distribution of *t*-test *P*-values (ranging from 0 to 1), and observed a relative reduction in *P*-values at the low end of the range (Figure 4H): if there were differences in the gene expression between the two groups, there should be a spike in *P*-values at the low end of the range. In summary, these three approaches suggest at the level of the PBMC transcriptome, there was actually little direct interaction between DEX and HA exposures.

### Interaction between sex and either DEX prophylaxis or HA exposure

**Figure 5:**
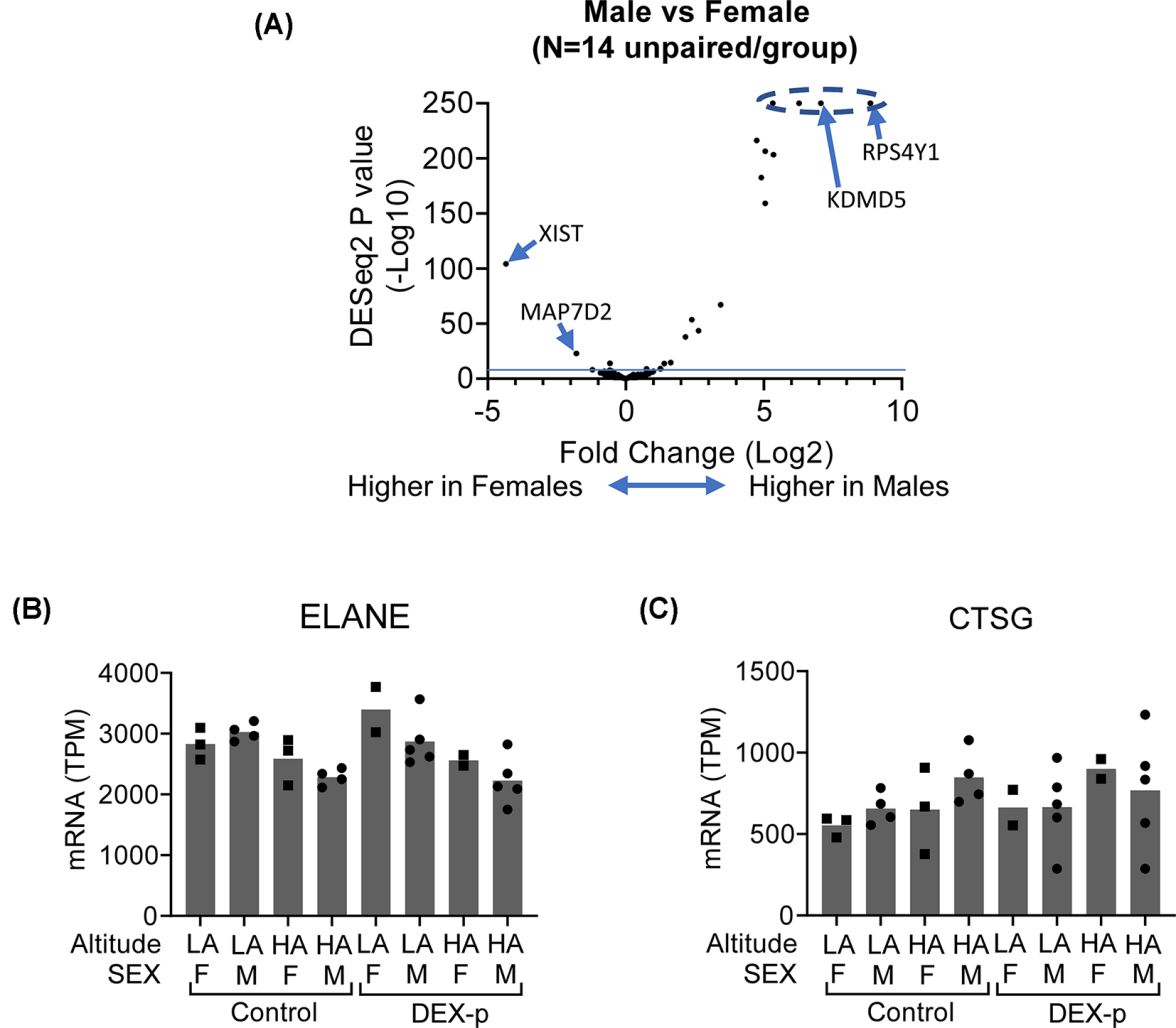
Little evidence of interaction between sex and either DEX prophylaxis or HA exposure (**A**) Volcano plot, comparing fold change on the *x*-axis (log base 2) versus *P*-value (−log base 10) by DESeq2 (*N* = 14/group); the four points in the dotted zone have *P*-value of zero (i.e.,−log_10_(*P*) = ∞). Representative expressions of two genes that were differentially expressed by both sex and DEX prophylaxis, (**B**) ELANE and (**C**) CTSG (mean shown; *N* = 2–5/group).

We compared the expression in the samples from the nine males versus the five females in our dataset. We found 50 sequences with a corrected *P*-value < 0.05, of which 21 had at least a 2-fold change either up or down-almost all of which were located on either the X or Y chromosome (Figure 5A). None of the 50 overlapped with any of the 2607 sequences differentially expressed by HA. Two of the 50 sequences overlapped with the 167 modulated by DEX prophylaxis: *ELANE* (encoding neutrophil elastase) and *CTSG* (encoding Cathepsin G, Figure 5B,C). The expression of both of these genes was slightly higher in females, and also higher in the DEX-p groups: a very modest synergistic effect between female sex and DEX prophylaxis.

### Deconvolution analysis to identify specific cell types

**Figure 6:**
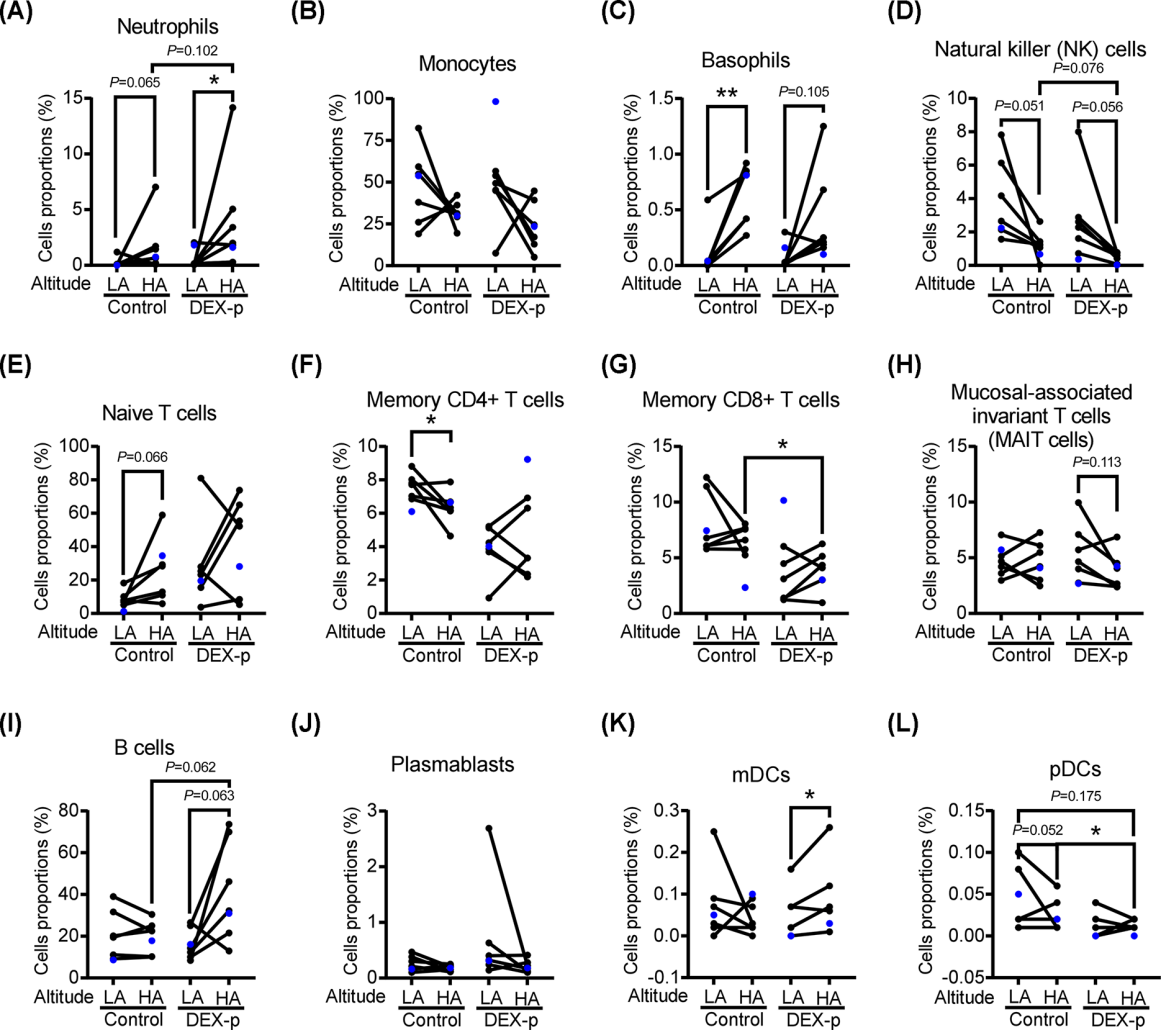
In-silico deconvolution analysis to identify the contribution of cell populations to the aggregate PBMCs (**A**) Neutrophils. (**B**) Monocytes. (**C**) Basophils. (**D**) Natural killer cells. (**E**) Naïve T cells. (**F**) Memory CD4+ T cells. (**G**) Memory CD8+ T cells. (**H**) Mucosal-associated invariant T cells. (**I**) B cells. (**J**) Plasmablasts. (**K**) Myeloid dendritic cells (mDCs). (**L**) Plasmacytoid dendritic cells (pDCs). Mean ±SD shown. Paired *t*-test (*N* = 6/group), *P<0.05; **P<0.01. Unpaired samples in each group (*n* = 1/group) have been shown in blue dots and were not included in the statistical analysis.

To gain insight into the potential contribution of specific cell types in the aggregate PBMC data, we performed deconvolution analysis on the bulk expression data from each sample, which computationally infers cell type proportions (Figure 6). Comparing the control HA and LA groups, we observed a decrease in memory CD4 T cells and plasmacytoid DCs (7.7% versus 6.3%, *P*<0.05; and 0.04% versus 0.02%, *P*=0.052, respectively), and an increase in basophils with HA exposure (Figure 6C,F,L). In the DEX-p group, there were trends towards an increase in B cells (16.1% versus 42.7%, *P*=0.063) and myeloid DCs (0.06% versus 0.1%, *P*=0.052), and a decrease in NK cells (3% versus 0.6%, *P*=0.076; Figure 6D,I,K) with HA exposure. Comparing the effect of DEX prophylaxis at HA, there was a relative decrease of CD8 T cells as well as NK cells (6.8% versus 4%, *P*<0.05; 1.3% versus 0.6%, *P*=0.076, respectively), and a relative increase in B cells (20.1% versus 42.8%, *P*=0.062) in the DEX-p group at HA compared with the control group at HA (Figure 6D,G,I). Overall, these data suggest that HA and DEX-p converge on modulation of inflammatory cells and pathways, affecting either the bone marrow production of lymphocytes and monocytes, or the uptake of these cells into the tissue. In particular, there was synergism of the two HA and DEX-p stimuli on increasing neutrophils and B cells, and decreasing NK cells and plasmacytoid DCs.

## Discussion

This study aims to uncover mechanisms linking peripheral blood characteristics with lung vascular pathobiology, paving the way for more effective therapeutic strategies for HA diseases and potentially PH, even when its origins are not associated with HA etiologies. Our work builds upon prior research in this field by employing an impartial transcriptomic approach to study PBMCs including phenotypes of these cells, potential signaling pathways, and distribution of cells in the population.

Significant changes in the PBMC transcriptome with HA challenge were observed, particularly related to either inflammation, or the regulation of cell division, translation, or transcription. Similar studies have been performed previously, including a cohort of 98 subjects who acutely travelled from sea level to 2835 m, which also observed increased expression in genes associated with translation and protein folding, and cell growth and apoptosis, as well as energy metabolism [28]. A smaller study compared the acute change at day 1 versus that at day 3, finding many of the genes were up-regulated at both timepoints [29], and there was also significant modulation of inflammatory pathways (at both timepoints) as we found here. There was also recently published single cell RNAseq of PMBCs from patients with chronic HA-induced PH, which identified an increase in monocyte density and interestingly a decrease in HIF1α at this late timepoint [30]. Markers of altered inflammation are similarly present in the peripheral blood of patients with idiopathic pulmonary arterial hypertension at the genetic and proteomic levels [31,32]. Signs of elevated inflammation persist in native highlander populations adapted to HA, as compared with lowlanders, including in humans and animals [33–35].

In contrast, the impact of DEX prophylaxis was relatively modest on the PBMC transcriptome, despite the DEX dose being relatively large. For this clinical indication, the range of DEX dose varies from 2 to 8 mg twice daily [36]: here, we used a mid-range dose of 4 mg twice daily. The effects of DEX on the PBMC transcriptome were largely related to the suppression of the immune system. The effect of DEX treatment on PBMC phenotype has also been studied using cytokine protein release by PBMCs [37,38], similarly demonstrating suppression of inflammatory pathways and specifically decreased cytokine release.

By PBMC transcriptome analysis alone, we observed little interaction between DEX and HA exposures, with no clear revealing of mechanisms by which DEX protects against HA exposure by this approach. There is a possibility that DEX may help maintain cellular integrity as a functional impairment of peripheral immune cells in chronic HA-induced PH patients has been reported [30]. DEX also reduces lymphocyte activation, and decreases cytokine and chemokine production [39]. Our deconvolution data did reflect differences in changes in the proportions of PBMC subpopulations, which suggests a potential mechanism underlying the benefit of DEX may have to do with differences in bone marrow production of cells, apoptosis/death of cells, or recruitment of cells into peripheral tissues.

The effect of DEX may also be mediated by different impacts on cells in the bloodstream versus bodily tissues, stemming from divergent cellular behavior and distribution in these compartments. Concentrations of DEX in plasma and tissues will differ due to tissue-specific uptake, metabolism, and clearance mechanisms. These differences can lead to unique physiological outcomes in each compartment and require future studies to investigate DEX’s pharmacokinetics and pharmacodynamics in plasma and tissues to understand its effects.

Other medications, most notably acetazolamide, are also clinically used in prophylaxis against HA illness. Acetazolamide inhibits carbonic anhydrase, leading to an increase in pCO_2_, which reflexively raises respiratory rate, thereby improving oxygenation [40], and also causing mild diuresis. The combination of acetazolamide and DEX may be particularly effective [41], but this needs to be studied further.

We suspect that DEX may modulate HA diseases by suppressing inflammation, as we found inflammation was increased by HA exposure, and suppressed by DEX prophylaxis. A key limitation of our analysis is that PBMC analysis does not reflect the phenotype of the lung tissue directly, and hypoxia is known to increase inflammation in the lungs [1–3]. Suppressing inflammation (as reflected in the PBMC transcriptome) or modifying the migration of cells out of the circulation and into the lung tissue may be critical modulators of lung inflammation. The deconvolution analysis suggests a number of cellular populations may be involved, including innate immune cells (dendritic cells, basophils, and monocytes) and adaptive immune cells (B and T cells). While it remains to be determined which are mechanistically causative versus those that are epiphenomenon, research has implicated a number of these populations in preclinical and clinical studies [42].

Other limitations of this work are the relatively small sample size, although the analysis of paired samples collected from the same subject at both LA and HA, does increase the statistical power. Our clinical cohort is relatively young, and the HA and DEX phenotype may be different in an older population. Similarly, our cohort was exclusively of Indo-Aryan ancestry, and the response to HA and DEX also can be different in other populations. This may explain differences between precise gene datasets in different studies, such as comparing our data to Pham et al., which enrolled subjects in California [29], although the general pathways modulated have a high degree of overlap. We only tested a single dose of DEX, and the effect may be more significant or additional pathways impacted at higher doses. This study also does not have a DEX-treated cohort that remained at LA, so we also cannot account for temporal changes following DEX prophylaxis that are independent of the exposure to HA. Deconvolution of bulk RNAseq data is becoming more common for identifying cell subpopulations, but is less accurate than direct cell counting through conventional methods such as flow cytometry.

In summary, we observed many significant changes in the PBMC transcriptome with HA exposure, and more modest changes in the transcriptome with DEX prophylaxis. There was little interaction in the PBMC transcriptome between concurrent HA and DEX exposures. There were changes in the potential cell populations within the deconvoluted bulk RNAseq data. These observations suggest that the mechanism by which DEX prophylaxis may mitigate the effects of HA exposure may be through blocking inflammation and modifying cell population numbers and locations.

## Data Availability

The data that support the findings of this study are available from the corresponding author upon reasonable request.

## Open Access

Open access for this article was enabled through a transformative open access agreement between Portland Press and the University of California.

## Abbreviations

ADAMTS2: A disintegrin and metalloproteinase with thrombospondin motifs 2 protein
CCNB2: cyclin B2
CCR2: C-C chemokine receptor type 2
CD4: cluster of differentiation
CEACAM8: CEA cell adhesion molecule 8
CTSG: cathepsin G
CX3CR1: C-X3-C motif chemokine receptor 1
CXCR4: C-X-C chemokine receptor type 4
DC: dendritic cell
DEX: dexamethasone
ELANE: elastase, neutrophil expressed
EPAS1: endothelial PAS domain protein 1
GLUT4: glucose transporter type 4
HA: high-altitude
HES4: hes family bHLH transcription factor 4
HIF1A: hypoxia inducible factor 1 subunit α
IFI27: interferon α inducible protein 27
IRF1: interferon regulatory factor 1
LA: low-altitude
LRRC26: leucine rich repeat containing 26
NK: natural killer cells
PBMC: peripheral blood mononuclear cell
PCA: principal component analysis
PDK1: pyruvate dehydrogenase kinase 1
PH: pulmonary hypertension
STAT1: signal transducer and activator of transcription 1
TSC22D3: TSC22 domain family member 3
UICLM: up-regulated in colorectal cancer liver metastasis
VSIG4: V-set and immunoglobulin domain containing 4 protein
